# Altered cytotoxicity of ROS-inducing compounds by sodium pyruvate in cell culture medium depends on the location of ROS generation

**DOI:** 10.1186/s40064-015-1063-y

**Published:** 2015-06-17

**Authors:** Jessica L Kelts, James J Cali, Sarah J Duellman, John Shultz

**Affiliations:** Department of Chemistry and Biochemistry, University of Michigan-Flint, 556 Murchie Science Building 303 E. Kearsley St., Flint, MI 48502 USA; Research and Development, Promega Corporation, 2800 Woods Hollow Dr., Madison, WI 53711 USA

**Keywords:** Cell culture, Hydrogen peroxide, Reactive oxygen species, Cytotoxicity

## Abstract

Induction of oxidative stress by drugs and other xenobiotics is an important mechanism of cytotoxicity. However, in vitro studies on the relationship between oxidative stress and cytotoxicity in cultured cells is frequently complicated by the fact that cell culture medium components affect reactive oxygen species (ROS) exposures in ways that vary with the mode of ROS production. The objectives of this study were to first determine the mode of ROS induction by certain model compounds when they are applied to cultured cells, and then to determine how ROS induction and cytotoxicity were affected by the ROS-quenching medium component pyruvate. Three compounds, eseroline, benserazide, and pyrogallol induced H_2_O_2_ in cell culture media independent of cells. However, another compound, menadione, induced H_2_O_2_ in a manner largely dependent on the MDA-MB-231 breast cancer cells used in this study, which is consistent with its known mechanism of inducing ROS through intracellular redox cycling. 1 mM pyruvate, as well as catalase, reduced the H_2_O_2_ in culture wells with each ROS inducer tested but it only reduced the cytotoxicity of cell-independent inducers. It reduced the cytotoxicity of benserazide and pyrogallol >10-fold and of eseroline about 2.5-fold, but had no effect on menadione cytotoxicity. From this data, it was concluded that depending on the mechanism of ROS induction, whether intra- or extracellular, a ROS-quenching medium component such as pyruvate will differentially affect the net ROS-induction and cytotoxicity of a test compound.

## Background

Modern toxicity testing places a strong emphasis on in vitro systems that employ cultured cells and various assay chemistries to deliver data that predicts in vivo outcomes (Niles et al. 2008). While cell viability and cell death assays reveal the potency of toxins, mechanistic assays uncover their modes of action. An important mechanism of chemical toxicity is the induction of oxidative stress through the production of excess reactive oxygen species (ROS) such as superoxide, H_2_O_2_, singlet oxygen, and hydroxyl radical (Schroeder et al. 2001; Yasuda et al. 2011; Liu et al. 2010; Park et al. 2012; Kawai et al. 2003). While in vitro assay systems measure the impact of a test compound on ROS levels, proper data interpretation requires an understanding of how and where ROS is created and of how the assay system affects ROS levels. For example, certain polyphenolic and other food derived anti-oxidants added to cell culture medium undergo redox cycling that produces H_2_O_2_ independent of cells (Halliwell 2008; Babich et al. 2009; Long and Halliwell 2009). In this case, H_2_O_2_-dependent cytotoxicity is from the outside in. Furthermore, cells have a significant capacity to eliminate ROS (e.g. superoxide dismutase eliminates superoxide, catalase and glutathione peroxidase eliminate H_2_O_2_), so lower ROS levels may be observed with cells compared to a cell free control.

In contrast to cell-independent ROS producers, some compounds stimulate excess ROS production by a cellular mechanism such as redox cycling to a semiquinone that is then reoxidized to the quinone by oxygen, forming superoxide (Thor et al. 1982). The superoxide and the hydrogen peroxide created from it by superoxide dismutase are created inside of the cell, so the ROS-dependent cytotoxicity originates from inside of the cell.

Cell culture medium itself can influence ROS levels. The common medium component riboflavin undergoes light-dependent reactions that generate ROS (Grzelak et al. 2001). For this reason medium can be a significant source of ROS that varies with the age of the medium and its storage conditions. On the other hand, sodium pyruvate, a common medium component, neutralizes H_2_O_2_ in a reaction that produces acetate, water, and carbon dioxide (Giandomenico et al. 1997; Long and Halliwell 2009). Indeed, pyruvate protects cells from H_2_O_2_ cytotoxicity so the potency of a ROS-dependent toxin may vary with medium pyruvate content, especially if the ROS is generated in the medium as opposed to inside the cell where it is sequestered away from pyruvate in the medium (Babich et al. 2009; Halliwell 2008; Long et al. 2007; Clement et al. 2002; Andrae et al. 1985).

In this study, we used model cytotoxic compounds to determine how ROS-dependent compound toxicity might vary with the mode of ROS-induction and the composition of cell culture medium. By comparing their capacity to induce H_2_O_2_ in the presence or absence of cultured cells, it was possible to categorize compounds as cell-dependent or cell-independent ROS inducers. We also showed dramatic effects of the common medium component sodium pyruvate on the cytotoxicity of cell-independent ROS inducers and its relative lack of effect on the toxicity of a largely cell-dependent ROS inducer. These studies serve to provide a model approach for profiling the cytotoxicity of ROS inducers.

## Results

**Figure 1:**
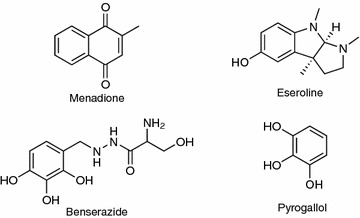
Structures of the ROS-producing compounds used in this study. The structures of menadione, eseroline, benserazide, and pyrogallol are shown.

We chose four known oxidants for this study (Figure 1) along with the pore forming peptide alamethecin to use as a control for ROS-independent cytotoxicity (Krauson et al. 2012). Menadione (Vitamin K3) is a cell-dependent ROS inducer that undergoes redox cycling in the cell, causing the accumulation of excess superoxide that is in turn converted to H_2_O_2_ by superoxide dismutase (Fukui et al. 2012; Buckman et al. 1993; Thor et al. 1982). Eseroline, benserazide, and pyrogallol were positive hits in a library screen for compounds that produce H_2_O_2_ in cell culture medium. Benserazide is used in conjunction with levodopa to treat Parkinson’s disease (Ossig and Reichmann 2013). Pyrogallol is a phenolic compound that produces superoxide and causes ROS-dependent organ damage in animals; it is a component of cytotoxic *Citrus aurantium* extracts (Karimi et al. 2012). Eseroline is an opioid agonist and analgesic drug (Furst et al. 1982). Benserazide and pyrogallol have multiple adjacent hydroxyl groups on a phenyl ring that mediate oxidation in cell culture medium (Long et al. 2010). Although eseroline does not appear to be an oxidant, it does form catechol and quinone breakdown products in medium that can produce ROS and this may account for its reported cytotoxicity against cultured neurons (Somani et al. 1990).

### ROS induction in medium

**Figure 2:**
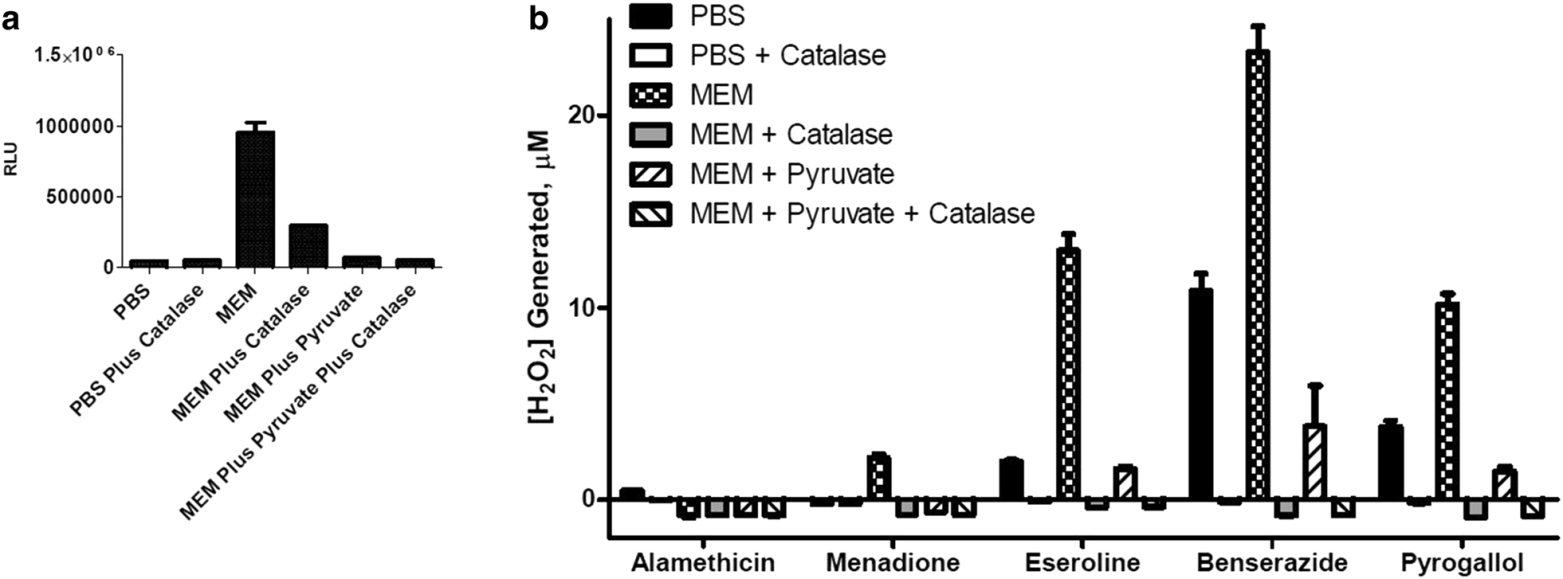
H_2_O_2_ in PBS and MEM. **a** PBS and MEM or **b** compounds (10 μM) were incubated plus or minus 1 mM sodium pyruvate (1 mM) or 35 U catalase at 37°C in a 5% CO_2_ incubator for 60 min in the presence of 25 μM H_2_O_2_ substrate. Measured luminescence is shown as relative light units (RLU) in part **a**. Measured luminescence was converted to concentration of H_2_O_2_ after background correction using a standard curve in part **b**. Data points are shown with *error bars* representing ± SEM. Data is representative of three separate experiments with triplicate determinations of each data point.

**Figure 3:**
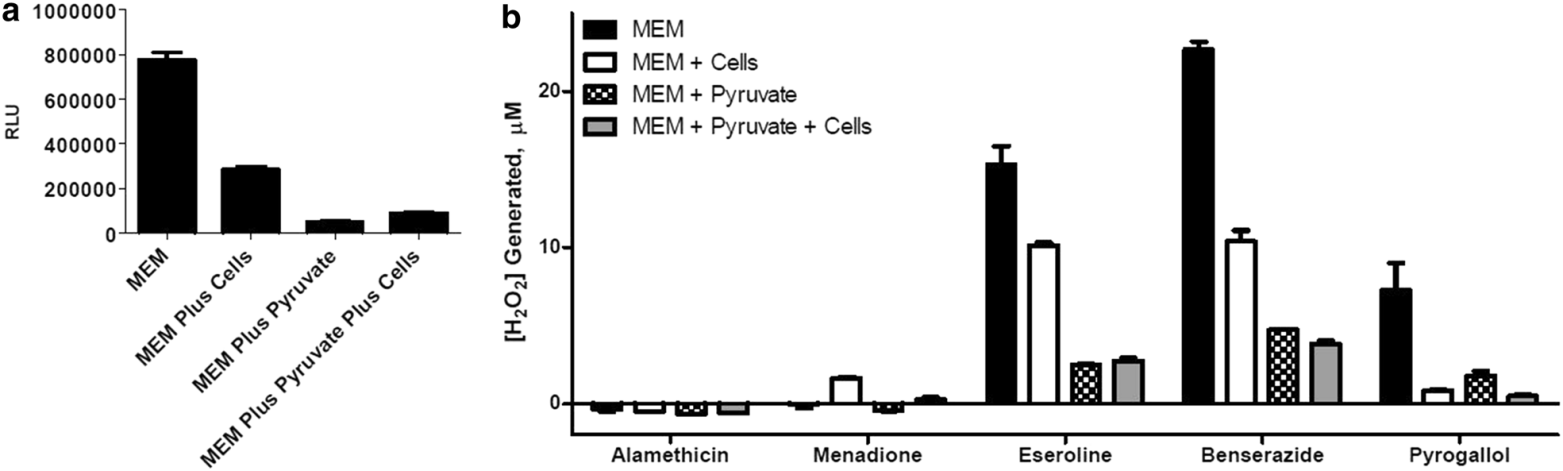
MDA-MB-231 cell effects on H_2_O_2_ levels. H_2_O_2_ was measured in MEM plus and minus 10,000 MDA-MB-231 cells and plus and minus 1 mM sodium pyruvate. **a** MEM or **b** 10 μM compounds were co-incubated with 25 μM H_2_O_2_ substrate for 60 min at 37°C in a 5% CO_2_ incubator. Measured luminescence is shown as relative light units (RLU) in part **a**. Measured luminescence was converted to concentration of H_2_O_2_ after background correction using a standard curve in part **b**. Data points are shown with *error bars* representing ± SEM. Data is representative of three separate experiments with triplicate determinations of each data point.

We examined the capacity of the selected compounds to produce ROS independent of cells by measuring H_2_O_2_ production in PBS or MEM plus and minus 1 mM sodium pyruvate, a ROS quencher that reacts with H_2_O_2_ to form acetate, H_2_O and CO_2_ (Long and Halliwell 2009), and/or 1 µg·ml^−1^ catalase, which converts H_2_O_2_ to O_2_ and H_2_O. Catalase and pyruvate substantially eliminated ROS assay luminescence, consistent with a correlation between [H_2_O_2_] and assay signals (Figure 2). Neat MEM presented a significant pyruvate- and catalase-sensitive H_2_O_2_ signal (Figure 2a). The modest catalase-insensitive signal from PBS and MEM apparently corresponds to non-specific assay background. Because the background for samples in MEM vs. PBS or MEM with additions were different, all data were background corrected before the amount of peroxide generated was quantitated using a standard curve. Values presented as slightly below zero in Figures 2 and 3 had values slightly below their corresponding background sample. The compound vehicles did not increase H_2_O_2_ signals in PBS or MEM (data not shown), nor did Alamethicin (Figure 2b). While menadione did not increase signals in PBS or MEM with pyruvate, it did cause a marginal increase in MEM. In contrast, eseroline, benserazide, and pyrogallol caused H_2_O_2_ signal increases in PBS and MEM with pyruvate and very large H_2_O_2_ signal increases in MEM. Catalase reduced signal to background levels, however a very large amount of catalase (35 U) had to be used for the experiment in MEM with pyruvate to reduce the levels to background (Figure 2b). Even though the amount of catalase needed to reduce signal to background was large, this shows that the probe used in this case is selective for H_2_O_2_. It is possible the large amount of catalase needed is because the large amount of anions in the media with pyruvate is inhibiting catalase. Catalase has been shown to be inhibited by anions, including acetate and formate (Agner and Theorell 1946). Pyruvate has the same carboxylate group as acetate and formate so it may have been able to partially inhibit the catalase. In addition, the reaction of pyruvate with peroxide in the media produces acetate.

### ROS induction and MDA-MB-231 cell cytotoxicity

The effect of MDA-MB-231 cells on H_2_O_2_ levels produced by the various compounds was determined by measuring H_2_O_2_ production in wells with 10,000 cells each. Luminescent values were background corrected before conversion to [H_2_O_2_] as above in experiments without cells. While cells enhanced H_2_O_2_ induction by menadione, they decreased H_2_O_2_ signal in MEM (Figure 3a) and decreased induction measured with eseroline, benserazide, and pyrogallol (Figure 3b). Furthermore, pyruvate reduced H_2_O_2_ signals for each treatment, with and without cells present.

**Figure 4:**
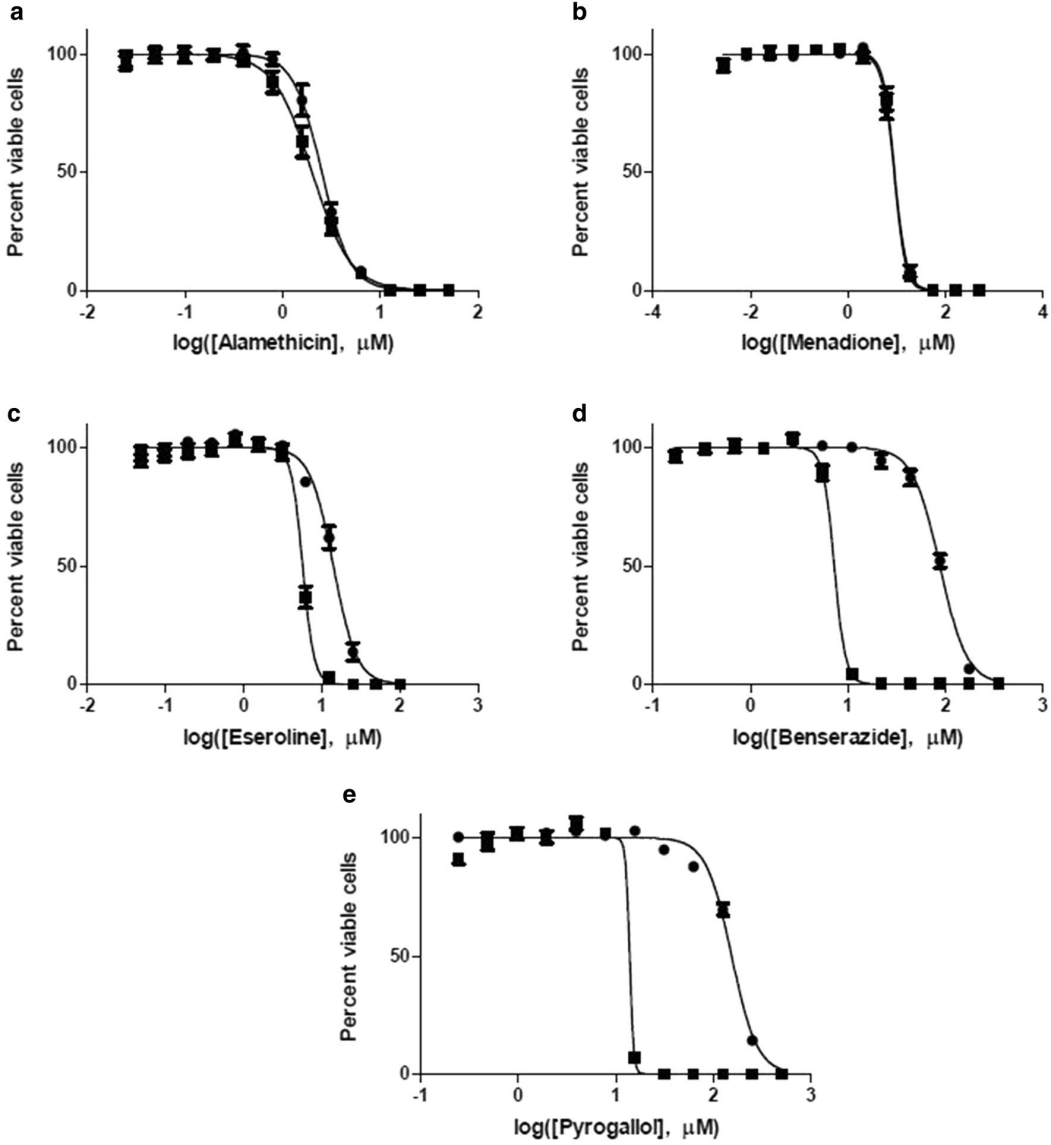
Effect of sodium pyruvate on cytotoxicty. MDA-MB-231 cells were incubated with increasing amounts of test compounds **a** alamethicin, **b** menadione **c** eseroline, **d** benserazide, or **e** pyrogallol for 68 h. Cell viability was determined by luminescent ATP detection. Results are shown as percent viable cells, mean ± SEM. Data is the average of three separate experiments with quadruplicate determinations of each point. *Filled square* MEM, *filled circle* MEM plus 1 mM pyruvate.

To examine the cytotoxicity of our test compounds and the impact of extracellular H_2_O_2_ quenching we measured cell viability after exposing MDA-MB-231 cells in MEM to a range of compound concentrations in the presence or absence of 1 mM sodium pyruvate. Pyruvate had minimal or no impact on the toxicity of alamethacin and menadione, which do not produce substantial ROS in MEM without cells (Figure 4). EC_50_ values express the toxic potency of each compound (Table 1).

**Table 1:** EC_50_ values for five test compounds with MDA-MB-231 cells

	Without pyruvate		With pyruvate	
	EC_50_ (μM)	95% confidence	EC_50_ (μM)	95% confidence
Alamethicin	2.0	1.9–2.2	2.5	2.4–2.7
Menadione	9.0	8.3–9.8	9.0	8.3–9.7
Eseroline	5.7	5.4–6.0	14.1	13.3–15.0
Benserazide	7.3	6.9–7.6	87.3	83.8–91.0
Pyrogallol	15.8^a^	None estimated^a^	153.6	148.1–159.2

^a^Due to the steepness of the cytotoxicity curve, there was high uncertainty in the fit for this compound such that a 95% confidence interval was not estimated

In contrast, pyruvate decreased the toxicity of the extracellular H_2_O_2_ inducers eseroline, benzerazide and pyrogallol by 2.5-, 12- and 11-fold, respectively (Figure 4; Table 1). Although a high degree of uncertainty is associated with the calculated EC_50_ of 13.8 μM for pyrogallol, it is a reasonable working number because it is near the midpoint of the curve.

## Discussion

In this study, we illustrated some basic parameters for identifying and characterizing ROS-inducers in cell culture. We chose to measure H_2_O_2_ because of its relative stability and ease of measurement compared to other ROS such as superoxide. The bioluminescent H_2_O_2_ assay we used was attractive because it incorporates a probe that reacts directly with H_2_O_2_, in contrast to systems that require horseradish peroxidase to catalyse the H_2_O_2_ reaction with a probe. Whereas a horseradish peroxidase-based system is highly vulnerable to small molecule interferences, the bioluminescent system is relatively impervious to such interferences (Duellman et al. 2013).

We measured H_2_O_2_ levels in the presence and absence of cells to determine if model compounds induce ROS in a cell-independent manner via intrinsic instability in culture medium, or by enhancing cell-dependent ROS production. Eseroline, benserazide, and pyrogallol induced H_2_O_2_ in a cell-independent manner, likely through redox cycling with medium components (Halliwell 2008; Babich et al. 2009; Long and Halliwell 2009). In contrast, ROS induction by menadione was largely cell-dependent, which is consistent with its known mechanism of ROS induction through mitochondrial disruption (Buckman et al. 1993; Fukui et al. 2012; Thor et al. 1982).

We also demonstrated the influence of medium composition on apparent compound cytotoxicity with the H_2_O_2_ quencher sodium pyruvate. This served to demonstrate that cell-independent ROS induction and its consequent cytotoxicity varies with medium composition (e.g. plus and minus pyruvate) while cell-dependent ROS induction associated with cytotoxicity is substantially insensitive to ROS quenching by medium. Furthermore, we suggest using pyruvate as a diagnostic tool whereby an observation of reduced compound cytotoxicity in the presence of pyruvate may indicate ROS-dependent toxicity via medium-dependent ROS induction.

We also observed that the commercially sourced MEM used for this study contained a significant amount of H_2_O_2_ without adding a ROS-inducer (Figures 2a, 3a). Previous reports indicate that H_2_O_2_ levels vary in common cell culture media and increase with exposure to light (Grzelak et al. 2001). It was tempting to speculate on how H_2_O_2_ in medium might cause an apparent enhanced cytotoxicity of an otherwise ROS neutral compound. Contrary to this concern, added sodium pyruvate did not reduce cytotoxicity of the presumed ROS neutral alamethicin. While this is inconsistent with an additive or synergistic effect of medium H_2_O_2_ and alamethecin, it does not preclude this possibility for other ROS neutral compounds. However, the cells themselves may mitigate this concern to some extent by reducing medium ROS as observed in this study (Figure 3a).

MDA-MB-231 cells in MEM without pyruvate reduced eseroline-, benserazide-, and pyrogallol-induced H_2_O_2_ levels as expected given the anti-oxidant capacity of living cells (Figure 3b). H_2_O_2_ diffuses into cells from the extracellular space, so enzymes such as catalase and glutathione peroxidase would have actively eliminated it (Bienert and Chaumont 2014; Bienert et al. 2006; Dringen and Hamprecht 1997; Dringen et al. 2005; Dunning et al. 2013; Venuprasad et al. 2013). The contrasting cell-dependent increase in H_2_O_2_ from menadione is also expected because menadione induces superoxide production by the mitochondria, which is then converted to hydrogen peroxide (Buckman et al. 1993; Fukui et al. 2012; Thor et al. 1982). In this case (Figure 3b), the rate of menadione-induced H_2_O_2_ production apparently outpaces the cells capacity to eliminate it.

It is not surprising that added pyruvate reduced cell-dependent ROS induction by menadione (Figure 3b) because we did observe a small H_2_O_2_ induction by menadione in MEM alone (Figure 2b) and because a portion of the intracellular H_2_O_2_ produced by mitochondrial uncoupling would diffuse into the medium and react with the pyruvate. Nevertheless, added pyruvate did not diminish menadione cytotoxicity indicating that extracellular H_2_O_2_ does not contribute to its toxic mechanism (Figure 4b; Table 1). It is reasonable to assume that intracellular H_2_O_2_ from redox cycling of menadione does contribute to its toxic mechanism and that this intracellular pool is protected from quenching by extracellular pyruvate.

Cell-dependent and cell-independent ROS inductions represent two fundamentally different mechanisms, each with its own implications for cytotoxicity. The cytotoxicity of cell-independent ROS-inducers like eseroline, benzerazide and pyrogallol should be more sensitive to extracellular ROS quenching than an intracellular ROS inducer such as menadione. Indeed, in this study pyruvate in culture medium reduced the cytotoxicity of eseroline, benzerazide and pyrogallol but not of menadione. The capacity of pyruvate to both quench extracellular H_2_O_2_ (Figures 2, 3) and reduce eseroline, benzerazide and pyrogallol cytotoxicity (Figure 4) suggests a correlation between the two effects that is consistent with at least a partial role for extracellular H_2_O_2_ production in their cytotoxic mechanism. Furthermore, the extent of right shift to the cytotoxicity curves by pyruvate was greater for benzerazide and pyrogallol than for eseroline, indicating a greater role for extracellular H_2_O_2_ in the cytotoxicity of the former two compounds compared to the latter.

## Conclusions

In vitro systems with cultured cells, such as the one used for this study, are valuable tools for identifying and characterizing compounds that induce ROS to levels that may damage cells. However, data retrieved from these systems can mislead if the mode of ROS induction is unknown and if the impact of cell culture medium on the magnitude of ROS induction is not recognized. Furthermore, certain discrepancies between different in vitro cytotoxicity studies may depend on the contrasting capacities of different media to enhance or protect against oxidative stress. The approach taken here illustrates how key parameters in cell-based ROS studies can be considered for developing mode of action profiles for ROS-inducing cytotoxins.

## Methods

All materials were reagent grade. Minimal essential media (MEM) Glutamax, 100X antibiotic/antimycotic, heat-inactivated FBS, 100 mM sodium pyruvate, and 0.25% Trypsin–EDTA were from Life Technologies (Grand Island, NY, USA). Alamethicin was from Cayman Chemical (Ann Arbor, MI, USA). DMSO, menadione, eseroline (fumarate salt), benserazide, pyrogallol, and catalase were from Sigma-Aldrich (St. Louis, MO, USA). CellTiter-Glo^®^ and ROS-Glo™ H_2_O_2_ Assays were from Promega Corp. (Madison, WI, USA).

### Cell culture

The human breast adenocarcinoma cell line MDA-MB-231 was obtained from ATCC (ATCC HTB-26). Cells were maintained in MEM with 10% heat-inactivated FBS, 100 U ml^−1^ penicillin, 100 µg·ml^−1^ streptomycin, 0.25 µg·ml^−1^ Fungizone^®^, and 1 mM sodium pyruvate. Cells were passaged twice per week and all data was generated between passages 6 and 30. Cells were passaged by washing once with phosphate buffered saline (PBS) and rinsing with 0.25% trypsin–EDTA solution. After dissociation from the cell culture flask, the cells were resuspended in MEM with or without pyruvate.

### Test compound treatments and ROS detection

We measured H_2_O_2_ production with the ROS-Glo™ H_2_O_2_ Assay system. All compounds were tested at 10 µM in PBS or MEM plus or minus 1 mM sodium pyruvate. Compounds were tested in triplicate and each experiment was performed a minimum of three separate times. Stock solutions were prepared as follows: 10 mM menadione in DMSO, 70 mM pyrogallol in DMSO, 40 mM benserazide in DMSO, 10 mM eseroline in DMSO, and 10.2 mM alamethicin in ethanol. Vehicle was present at 0.1% in these experiments. The H_2_O_2_ assay was performed as directed by the manufacturer as follows. The compounds were added to an opaque white 96 well plate in the desired media or PBS. The H_2_O_2_ substrate solution was then added, bringing the final volume to 100 µl. The plate was incubated at 37°C in a 5% CO_2_ incubator for 60 min. 100 µl of the ROS-Glo detection solution was added to each well at the end of the incubation. After an additional 20 min incubation at room temperature, luminescence was recorded using a GloMax^®^ Multi Detection System luminometer.

To confirm the selectivity of ROS-Glo for H_2_O_2_, 35 U of catalase was included in some of the 100 μl reactions. In order to estimate the amount of H_2_O_2_ generated by these compounds, a standard curve was also performed at the same time as the corresponding experiment. Concentrations between 0 and 30 μM H_2_O_2_ were used to construct the standard curve. A standard curve in MEM media was used to quantitate the data using MEM media or MEM with pyruvate and a standard curve in PBS was used to quantitate the data in PBS. Due to the large difference in background between samples in MEM, samples in MEM with pyruvate, and those containing catalase, all data points and the standard curve were background corrected using the appropriate controls. The equation of the linear line of the curve was then used to determine the amount of H_2_O_2_ generated in each sample.

Assays to determine the production of H_2_O_2_ in the presence of cells were performed as described above with the following modifications. The day before the experiment, cells were plated in clear, tissue culture treated 96-well plates at a density of 10,000 MDA-MB-231 cells per well in a total volume of 70 µl. Wells with cells were paired with corresponding wells without cells (medium alone). After overnight incubation at 37°C in a 5% CO_2_ incubator, 10 µl of compounds or their vehicle in the appropriate medium were added, followed by 20 µl of the H_2_O_2_ substrate solution for a total volume of 100 µl. After the 1 h incubation at 37°C in a 5% CO_2_ incubator, 50 µl of each reaction mixture was transferred to a white 96-well plate and mixed with 50 µl of the ROS-Glo detection solution. The plate was then incubated 20 min at room temperature before reading luminescence. The standard curve for these experiments was performed analogously to the experiment. Briefly, the incubation of the H_2_O_2_ samples occurred in a cell culture treated plate, after which 50 µl was moved to a white luminometer plate and mixed with 50 µl of detection reagent.

### Cell viability measurements

MDA-MB-231 cells were plated at a density of 1,000 cells per well in 70 µl the day before cytotoxicity treatments in clear, cell culture treated 96-well plates. After overnight incubation at 37°C in a 5% CO_2_ incubator, 30 µl of test compound in the desired media was added to each well. Compounds were diluted two fold across a 12-well reagent reservoir before addition to the plate. The concentration of vehicle (either DMSO or ethanol) was 1% or less in cytotoxicity experiments and was the same in all wells of each plate. The plates were returned to the incubator for 68 h. Then the number of live cells remaining was determined as a function of ATP content using the CellTiter-Glo^®^ luminescent assay as directed by the manufacturer as follows. 100 µl reconstituted assay reagent was added to each well. The plate was agitated for 2 min on a plate shaker and incubated at room temperature for at least 15 min. 100 µl of the mixture was then transferred to a white 96 well plate and the luminescence was measured on a GloMax^®^ Multi Detection System luminometer.

Data points in each cell viability curve are the mean of twelve replicates obtained during three separate experiments. Data was analyzed using GraphPad Prism 5. Each individual curve was fit to a sigmoidal dose response curve. The value of the top plateau of the curve was used as the 100% value and all data points were expressed as a percentage of this value. Error bars indicate the standard error of the mean.

## Abbreviations

ROS: reactive oxygen species
EC_50_: concentration at half-maximal toxicity
PBS: phosphate buffered saline
MEM: minimal essential media
DMSO: dimethyl sulfoxide
SEM: standard error of the mean
